# Loss of secretin results in systemic and pulmonary hypertension with cardiopulmonary pathologies in mice

**DOI:** 10.1038/s41598-019-50634-x

**Published:** 2019-10-02

**Authors:** Aung Moe Zaw, Revathi Sekar, Sarah O. K. Mak, Helen K. W. Law, Billy K. C. Chow

**Affiliations:** 1 School of Biological Sciences, The University of Hong Kong, Hong Kong, China; 2 Department of Health Technology and Informatics, Faculty of Health and Social Sciences, The Hong Kong Polytechnic University, Hong Kong, China

**Keywords:** Cardiovascular biology, Experimental models of disease

## Abstract

More than 1 billion people globally are suffering from hypertension, which is a long-term incurable medical condition that can further lead to dangerous complications and death if left untreated. In earlier studies, the brain-gut peptide secretin (SCT) was found to be able to control blood pressure by its cardiovascular and pulmonary effects. For example, serum SCT in patients with congestive heart failure was one-third of the normal level. These observations strongly suggest that SCT has a causal role in blood pressure control, and in this report, we used constitutive SCT knockout (SCT^−/−^) mice and control C57BL/6N mice to investigate differences in the morphology, function, underlying mechanisms and response to SCT treatment. We found that SCT^−/−^ mice suffer from systemic and pulmonary hypertension with increased fibrosis in the lungs and heart. Small airway remodelling and pulmonary inflammation were also found in SCT^−/−^ mice. Serum NO and VEGF levels were reduced and plasma aldosterone levels were increased in SCT^−/−^ mice. Elevated cardiac aldosterone and decreased VEGF in the lungs were observed in the SCT^−/−^ mice. More interestingly, SCT replacement in SCT^−/−^ mice could prevent the development of heart and lung pathologies compared to the untreated group. Taken together, we comprehensively demonstrated the critical role of SCT in the cardiovascular and pulmonary systems and provide new insight into the potential role of SCT in the pathological development of cardiopulmonary and cardiovascular diseases.

## Introduction

Hypertension is a long-term medical condition that affects more than 1 billion people globally^1^ and ranks in the top position of the worldwide causes of death^2^. It is commonly referred to as systemic hypertension (SHT) and indicates high blood pressure in arteries from the heart, where the systolic blood pressure (SBP) is equal to or above 140 mmHg and/or diastolic blood pressure (DBP) is equal to or above 90 mmHg in humans^3^. Apart from SHT, pulmonary arterial hypertension (PAH) has a lower prevalence but a higher mortality rate in overall hypertensive patients^4,5^. PAH indicates high blood pressure particularly in the lungs caused by cellular proliferation and fibrosis of the small pulmonary arteries^6^. To maintain a stable blood pressure, a sophisticated biological system and other organ conditions are involved and closely related, and dysregulation of these systems or related organ failure, for example, congestive heart failure, portal hypertension^7^ or dysregulation of the renin-angiotensin-aldosterone system (RAAS)^8–10^, will lead to hypertension.

Secretin (SCT), a classical brain-gut peptide, has long been shown to have a functional role in blood pressure control due to its previously reported cardiovascular and pulmonary effects. Secretin receptor (SCTR) transcripts were found to be highly expressed in both the heart and lungs^11,12^. In the heart, SCT is able to reduce blood pressure^13^, increase cardiac blood flow, regulate myocardial contraction and control coronary vasodilation through endothelial release of nitric oxide (NO)^14–16^. In the lungs, SCT can stimulate chloride (Cl^−^) efflux from bronchial epithelial cells, which is important for airway surface liquid (ASL) and mucociliary clearance and tertiary bronchiole relaxation in humans^12^. In addition, SCT is found to be able to upregulate vascular endothelial growth factor (VEGF) expression in liver cholangiocytes to stimulate biliary cell proliferation^17^. Our previous studies have also shown that the SCT/SCTR axis can mediate the central action of angiotensin II (ANGII) and participate in body water and salt homeostasis by regulating vasopressin and aldosterone release^18–20^. More importantly, low SCT, NO and VEGF levels and significantly high aldosterone levels were found in patients with congestive heart failure^21,22^. Taken together, these data indicate that SCT has a close relationship with blood pressure control modulators (i.e., NO, VEGF, and aldosterone) and exhibits certain functions in the cardiovascular and pulmonary systems; however, no studies have clearly shown the significance of SCT in these systems and explained how SCT plays a role in regulating blood pressure. In this article, we investigated the severity of SCT deficiency on the cardiovascular and pulmonary system as well as the SCT-NO-VEGF-aldosterone mechanism using SCT^−/−^ mice. We comprehensively showed that loss of SCT can cause systemic and pulmonary hypertension as well as fibrosis in the heart and lungs. Clinical signs of congestive heart failure were also found in SCT^−/−^ mice with lower NO and VEGF and higher aldosterone levels in blood compared to control mice. Finally, we examined the possibility of using SCT as a novel therapeutic agent to treat hypertension and prevent the development of pathologies in the lungs and heart of hypertensive patients.

## Results

### Loss of SCT results in systemic hypertension and cardiac remodelling

**Figure 1:**
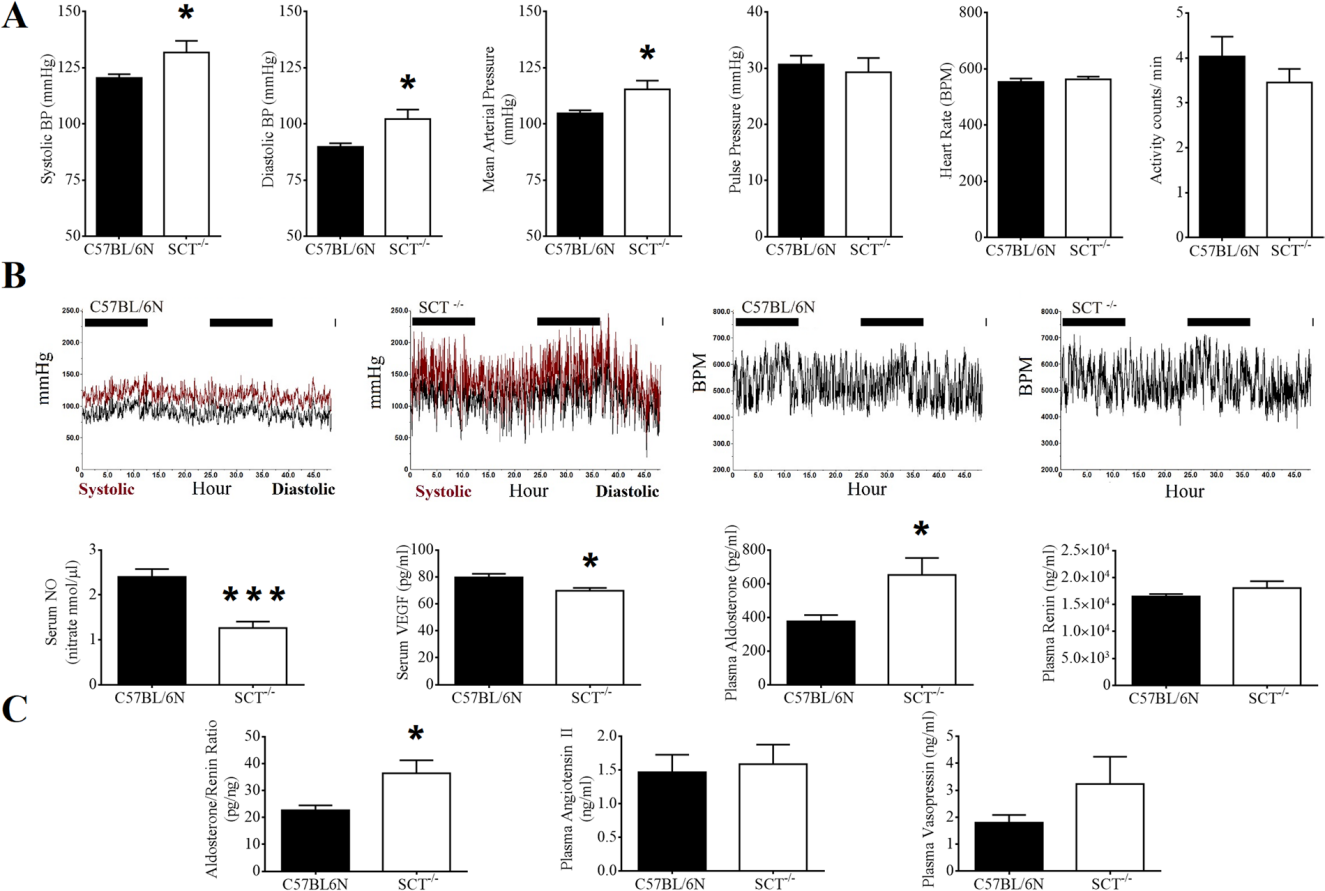
Systemic hypertension in SCT^−/−^ mice with related blood hormone level changes. (**A**) Systolic, diastolic and mean arterial pressures were significantly increased in SCT^−/−^ mice compared with 6‐month‐old C57BL/6N mice. The pulse pressure, heart rate, and activities were not significantly different (n = 12/group; *p < 0.05). (**B**) Representative 48-hour long systolic and diastolic blood pressure and heart rate tracings from 6‐month‐old C57BL/6N and SCT^−/−^ mice. The heart rate patterns were similar, but high blood pressure tracing was observed in SCT^−/−^ mouse. (**C**) NO (n; C57BL/6N = 10, SCT^−/−^ = 12) and VEGF (n = 8/group) levels were significantly decreased in SCT^−/−^ mice (*p < 0.05; **p < 0.01; ***p < 0.001). Significantly increased *p*lasma aldosterone and aldosterone to renin ratio with slightly increased plasma renin, angiotensin II, and vasopressin levels in SCT^−/−^ compared with C57BL/6N mice. (n = 7/group; *p < 0.05; **p < 0.01; ***p < 0.001).

To detect hypertension in SCT^−/−^ mice, cardiovascular parameters were measured continuously in freely moving healthy SCT^−/−^ mice by implantable telemetry. After 48 hours of continuous measurement, SBP, DBP and mean arterial pressure (MAP) were found to be significantly higher in 6-month-old SCT^−/−^ mice compared with the same age of wild-type mice (SBP: 138.91 ± 7.36 *vs*. 117.42 ± 2.96 mmHg, *p* = 0.0283; DBP: 110.25 ± 7.71 *vs*. 88.16 ± 1.89 mmHg, *p* = 0.0247, MAP: 122.81 ± 7.14 *vs*. 102.37 ± 2.31 mmHg, *p* = 0.0275; n = 12/group), while no difference was shown in the pulse pressure, heart rate and animal activity (Fig. 1A). Meanwhile, high SBP and DBP tracings were also observed in 48-hour-long blood pressure tracings (Fig. 1B).

**Figure 2:**
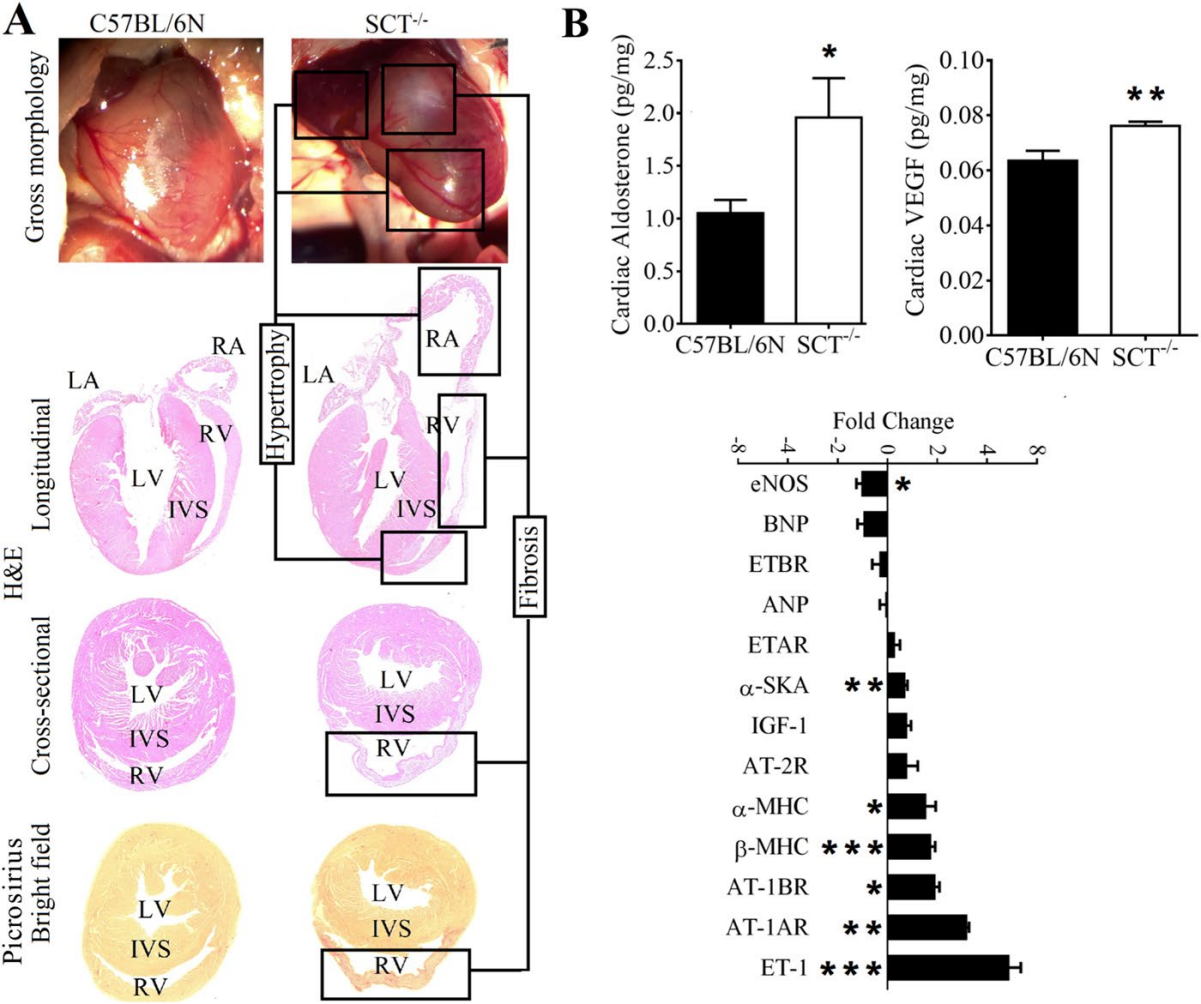
Cardiac remodeling, fibrosis, and related hormone and gene expression changes in SCT^−/−^ mice. (**A**) The gross morphology, H&E and Picrosirius Red Collagen staining of the hearts. The whitish fibrous tissue and deformed RV contour can be seen in the gross morphology of the heart of SCT^−/−^ mouse. The dilated RA with hypertrophic wall and fibrosis and hypertrophy mixed RV wall can be seen in the longitudinal section of the heart of SCT^−/−^ mouse. The deformed and dilated RV contour can be seen in the cross-sectional view of the heart of SCT^−/−^ mouse. In the picrosirius staining, the fibrous tissues can be seen as deep red fibers under bright field. (**B**) Increased cardiac aldosterone level and VEGF in SCT^−/−^ mice (n = 8/group; *p < 0.05, **p < 0.01). The gene transcript levels in the heart of SCT^−/−^ mice compared with C57BL/6N mice with internal control: GAPDH expression; 6-month-old mice; n = 6–7; *p < 0.05; **p < 0.01; ***p < 0.001 (n = 6/group for staining).

Next, the heart pathologies in SCT^−/−^ mice were identified by heart weight ratio measurements, gross morphology and histology analysis, as well as cardiac functional analysis by echocardiography. First, the right ventricle to left ventricle plus septum [RV/(LV + S)] ratio, an indicator of RV hypertrophy, heart to body weight and heart to tibia length ratios were significantly increased in 3-month-old SCT^−/−^ mice (Supplementary Fig. S1). However, these ratios were not relevant at 6 months or older ages of SCT^−/−^ mice because they have already developed RV deformation and fibrosis with myocardial tissue loss and hypertrophy. Second, although the upper part of the RV of SCT^−/−^ mice was dilated and fibrotic, the lower portion of the RV was also hypertrophied (Fig. 2A and Supplementary Videos S1 and S2). The left ventricular (LV) mass was found to be smaller in SCT^−/−^ mice by high frequency echocardiographic examination (Supplementary Table S3). Collectively, these measurements indicated that SCT^−/−^ mice have myocardial tissue loss in the LV and RV hypertrophy and fibrosis.

### Molecular changes in cardiac tissue hormones and protein in SCT^−/−^ mice

To identify the underlying mechanisms of SCT, NO, VEGF, aldosterone and other molecules affecting blood pressure and related to SCT were measured. In plasma, NO (1.26 ± 0.14 *vs*. 2.4 ± 0.17 nmol nitrate; *p* < 0.001) and VEGF (69.69 ± 1.96 *vs*. 79.7 ± 2.48; *p* < 0.05) levels were significantly reduced in SCT^−/−^ mice. The plasma aldosterone level (652.53 ± 92.74 *vs*. 376.68 ± 34.51 pg/ml; *p* < 0.05) and aldosterone to renin ratio (36.34 ± 4.44 *vs*. 22.68 ± 1.66 pg/ng; *p* < 0.05) were significantly higher. The plasma renin, ANGII and vasopressin levels were slightly higher in SCT^−/−^ mice, but there was no statistical significance (Fig. 1C). Furthermore, cardiac tissue aldosterone (1.05 ± 0.11 *vs*. 1.95 ± 0.35 pg/mg; n = 8/group; *p* = 0.0372) and cardiac VEGF (0.06 ± 0.00 *vs*. 0.07 ± 0.00 pg/mg; n = 8–9/group; *p* = 0.0085) were also significantly increased in SCT^−/−^ mice (Fig. 2B). In gene expression analysis in the heart of SCT^−/−^ mice, endothelin-1 (ET-1) transcript was observed together with a significant fold increase in ANGII type 1a receptor (AT-1AR), ANGII type 1b receptor (AT-1BR), and β- and α-myosin heavy chain (MHC) genes. However, endothelial nitric oxide synthase (eNOS) expression was reduced (Fig. 2B).

### Loss of SCT also leads to pulmonary arterial hypertension and histopathological changes in the lungs

**Figure 3:**
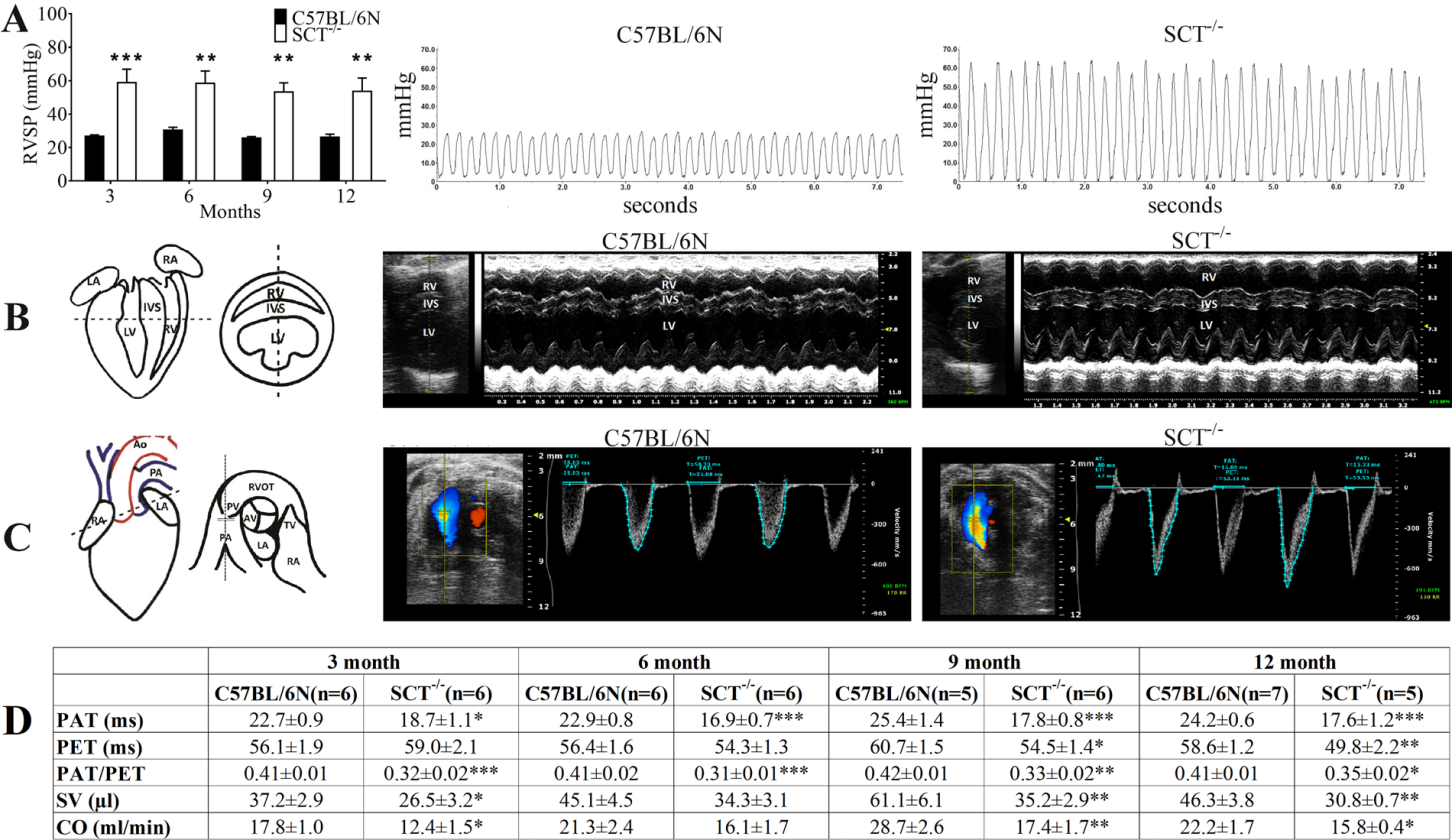
Pulmonary arterial hypertension in SCT^−/−^ mice. (**A**) RVSP was significantly increased in SCT^−/−^ mice than C57BL/6N mice in all experimental ages (*p < 0.05; **p < 0.01; ***p < 0.001). Representative RVSP waveforms from 6-month-old mice showed the two-fold increase. (**B**) Representative sketch of the bi-ventricular level echocardiographic plane and two‐dimensional (B-mode) and M‐mode echo images indicating RV dilation, wall thinning and thickened IVS in 6‐month‐old SCT^−/−^ compared with C57BL/6N. (**C**) Representative sketch of the aortic valve level echocardiographic plane, color Doppler and pulsed waveforms of 6-month-old mice. The reduction of PAT length and the slope of the wave in SCT^−/−^ is steeper than that of C57BL/6N mouse. [LA-Left Atrium, RA-Right Atrium, LV-Left Ventricle, IVS-Inter Ventricular Septum, RV-Right Ventricle, Ao-Aorta, PA-Pulmonary Artery, TV-Tricuspid Valve, RVOT-Right Ventricular Outflow Track, PV-Pulmonary Valve, AV-Aortic Valve, PAT-Pulmonary Acceleration Time, PET-Pulmonary Ejection Time, VTI-Velocity Time Integral]. (**D**) The PAT, PET, and PAT to PET ratio, RV stroke volume (SV) and RV cardiac output (CO) were reduced in SCT^−/−^ mice compared with the control group (*p < 0.05; **p < 0.01; ***p < 0.001).

Apart from SHT, we also studied the blood pressure parameters in the pulmonary circulation and echocardiographic parameters related to pulmonary circulation and right ventricular function to examine whether SCT^−/−^ mice exhibit PAH. The right ventricular systolic pressure (RVSP), which can reflect the pressure in the pulmonary circulation, was significantly increased in SCT^−/−^ mice of all age groups [3 months: 26.74 ± 0.81 (n = 8) *vs*. 58.84 ± 8.03 (n = 7) mmHg, *p* = 0.0009; 6 months: 30.37 ± 1.65 (n = 7) *vs*. 58.42 ± 6.83 (n = 7) mmHg, *p* = 0.0031; 9 months: 25.63 ± 0.86 (n = 6) *vs*. 53.26 ± 5.13 (n = 8) mmHg, *p* = 0.0011; 12 months: 26.2 ± 1.62 (n = 6) *vs*. 53.65 ± 7.23 (n = 6) mmHg, *p* = 0.0070] at ~350 bpm heart rate (Fig. 3A). In echocardiography, thickened RV walls were observed in 3-month-old SCT^−/−^ mice denoting early hypertrophy, while dilated RVs with thin free walls and thickened interventricular septum (IVS) were observed in SCT^−/−^ mice at 6 months of age and older (Fig. 3B and Supplementary Material Videos S1 and S2). The indices of pulmonary hypertension, i.e., PAT/PET (pulmonary acceleration time to pulmonary ejection time ratio) and PAT, the right ventricle (RV) stroke volume (SV) and cardiac output (CO) were reduced in SCT^−/−^ mice (Fig. 3C,D). While patent ductus arteriosus (PDA) was observed in a few 3-month-old SCT^−/−^ mice, these mice were excluded from the study. These data suggested that during the progression of PAH in SCT^−/−^ mice, 3-month-old SCT^−/−^ mice exhibited the initial stage of PAH with compensatory RV hypertrophy, but while the SCT^−/−^ mice progressed to RV dilation by 6 months, overall RV function, including SV and CO, was still maintained until 12 months of age. Collectively, these data showed that SCT deficiency causes moderate pulmonary arterial hypertension along the developmental state of the animal.

**Figure 4:**
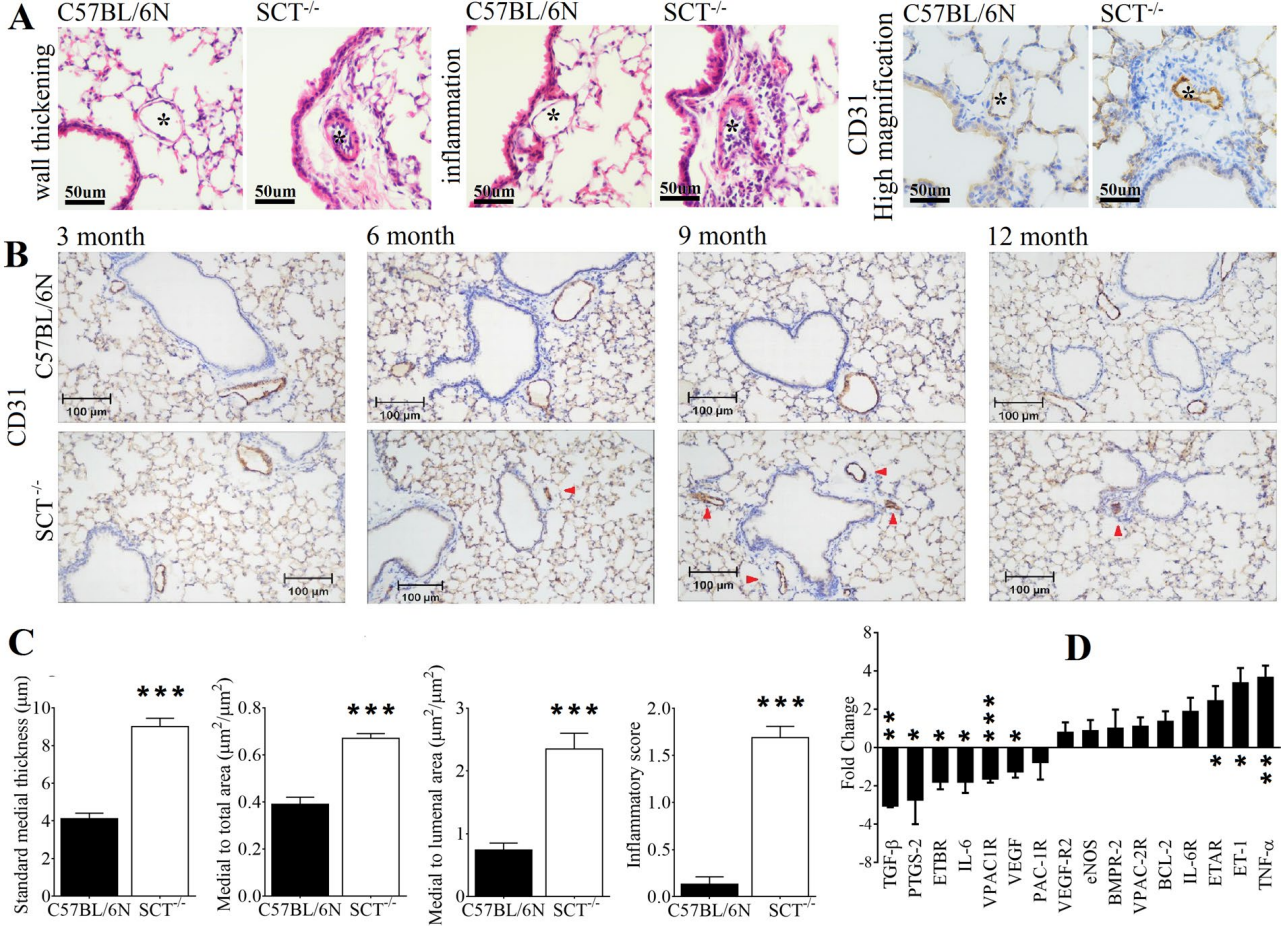
Arterial wall remodeling, perivascular inflammation, endothelial cell proliferation and gene expression changes in the lungs of SCT^−/−^ mice. * indicates lumen of an artery. (**A**) Arterial wall thickening, perivascular inflammation, and endothelial cell proliferation (CD31 staining) in 6-month-old SCT^−/−^ mice (n = 6/group). (**B**) CD31 immunohistochemical staining shows vascular remodeling in the lungs of SCT^−/−^ mice at 3, 6, 9 and 12 months. Red arrows indicate thickened arteries stained with CD31. (**C**) The ratios related to the arterial wall thickening and inflammatory scoring were significantly increased in 6-month-old SCT^−/−^ mice (n = 6/group; *** *p* < 0.001). (**D**) Gene transcript levels in the lungs of SCT^−/−^ mice compared with C57BL/6N mice with internal control GAPDH expression in 6-month-old mice (n = 6; *p < 0.05; **p < 0.001; ***p < 0.001).

Next, from the histopathological analysis of the lungs of SCT^−/−^ mice, arterial wall thickening and perivascular inflammation were observed. From the H&E staining of all experimental ages of SCT^−/−^ mice, the walls of the pulmonary arteries were thickened significantly compared to wild-type mice. When ~50-µm-diameter arteries were compared between 6-month-old control and SCT^−/−^ mice, significantly increased medial width (standard medial thickness) was found in SCT^−/−^ mice (9.01 ± 0.44 µm *vs*. 4.12 ± 0.29 µm; *p* < 0.001). The medial to total area ratio (0.67 ± 0.02 *vs*. 0.39 ± 0.03; *p* < 0.001) and medial to luminal area ratio (2.35 ± 0.25 *vs*. 0.74 ± 0.11; *p* < 0.001) were significantly elevated in SCT^−/−^ mice, indicating arterial wall thickening in the lungs of SCT^−/−^ mice (Fig. 4C and Supplementary Fig. S2). We also studied the local microvasculature of the lungs of SCT^−/−^ mice by immunohistochemical (IHC) staining against cluster of differentiation 31 (CD31). This protein is commonly used to demonstrate the presence of endothelial cells (EC) and help to evaluate the degree of angiogenesis^23^. In CD31 staining, the red arrows indicate thickened walls and narrowed lumens in the lungs of SCT^−/−^ mice, showing severe vascular remodelling of the arteries in the lungs of SCT^−/−^ mice notably from 6 months of age (Fig. 4A,B). Next, the inflammatory score of SCT^−/−^ mice was markedly higher than that of control (Fig. 4C), and a large number of eosinophils were found inside the lumen of pulmonary arteries and surrounding the respiratory ducts and alveoli from the H&E staining but were absent in wild-type mice (Fig. 4A). Because inflammation can activate a cascade of cellular and molecular events, such as activation of vascular cells, production of chemoattractant and recruitment of adhesion molecules^24^, these events will induce attachment of blood-borne inflammatory cells such as neutrophils and eosinophils^24,25^. However, despite the histological signs of inflammation in the lung sections, the observed luminal narrowing with CD31 staining may also be due to the endothelial cell proliferation, therefore we suggested that there was intimal hyperplasia in the lungs of SCT^−/−^ mice.

**Figure 5:**
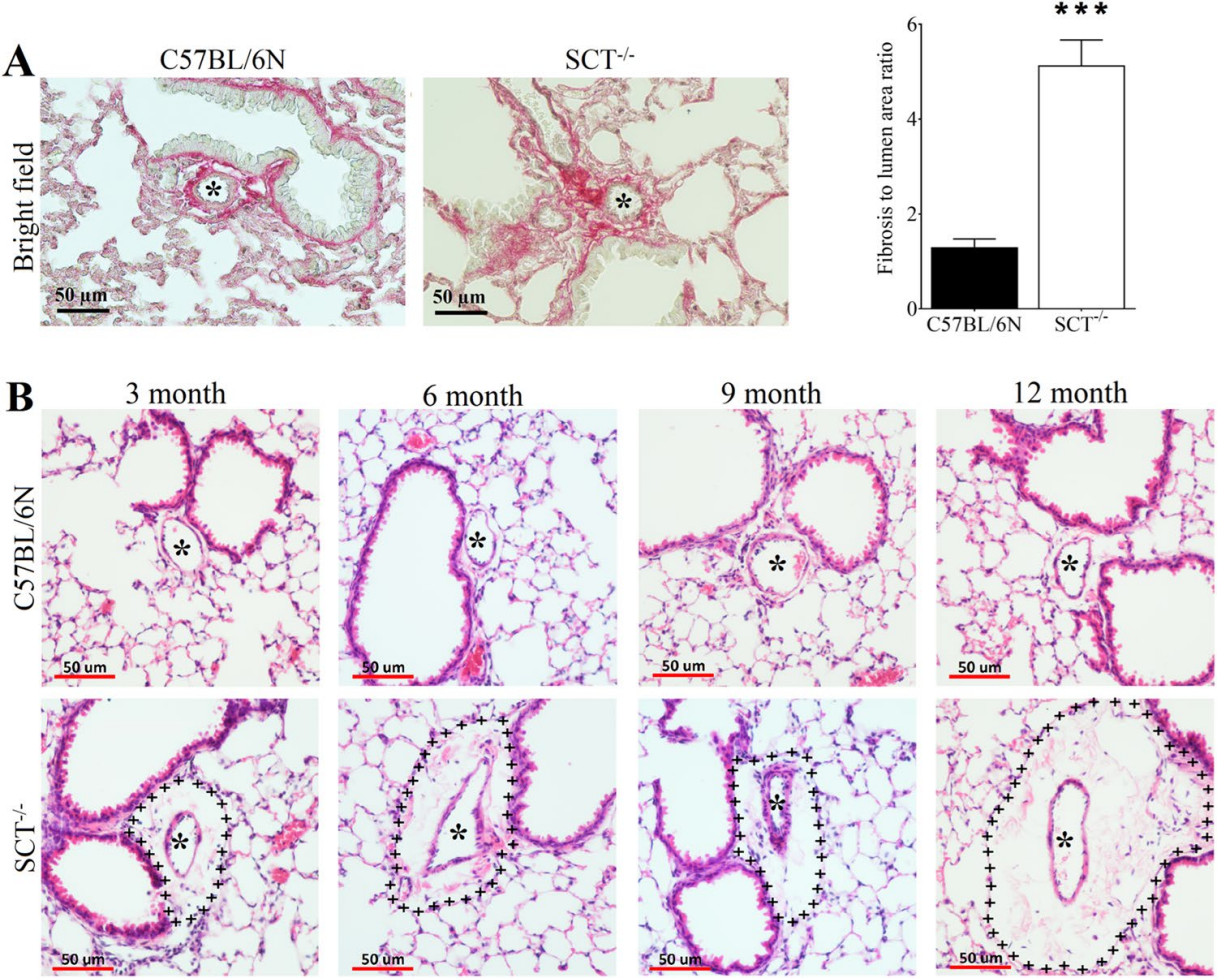
Perivascular fibrosis and adventitial expansion in the lungs of SCT^−/−^ mice. (**A**) Picrosirius red staining shows increased perivascular fibrosis as deep red fibers in SCT^−/−^ mice under bright field. The fibrosis to lumen area ratio is increased in 6-month-old SCT^−/−^ mice. (**B**) Representative figure of the perivascular adventitial expansion (perivascular space) in the lungs of 3,6,9 and 12-month-old SCT^−/−^ mice compared with same age WT mice. The (+++) line represents the adventitial expansion area (n = 6/group).

In addition, perivascular fibrosis and adventitial expansion were also found in the lungs of SCT^−/−^ mice. To detect fibrosis in the lung sections, picrosirius red collagen staining was performed in which the collagen fibres were revealed as a deep red colour under bright light (Fig. 5A). From the staining, substantially increased perivascular fibrosis was observed in 6-month-old SCT^−/−^ mice when compared with same age control mice. In addition, the fibrosis to lumen area ratio was increased in 6-month-old SCT^−/−^ mice compared to that in control mice (Fig. 5A). In the H&E staining, the perivascular space in all experimental ages of SCT^−/−^ mice was much larger than that in control mice, demonstrating that perivascular adventitial expansion was present in the lungs of SCT^−/−^ mice (Fig. 5B).

**Figure 6:**
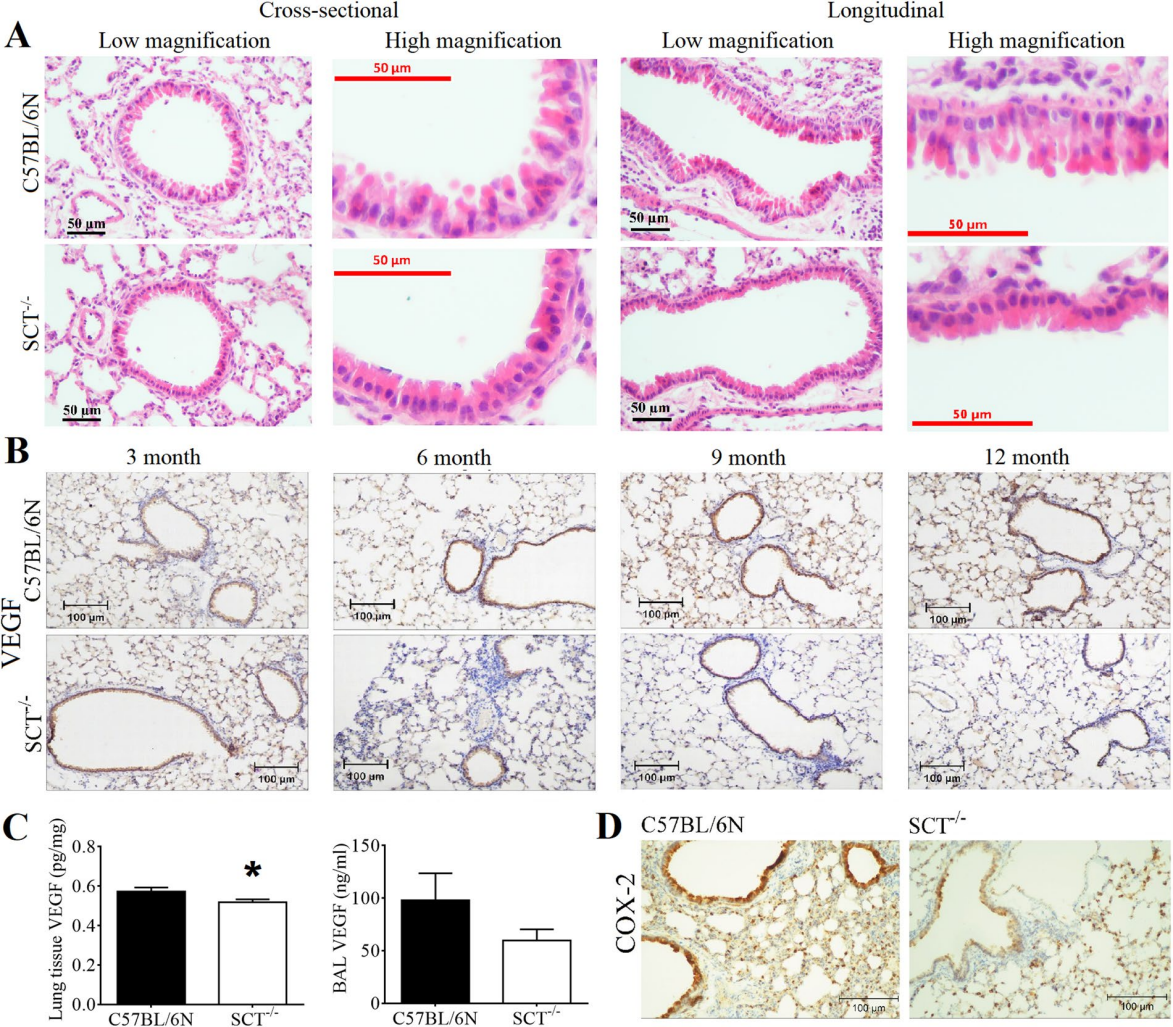
Small airway remodeling, and VEGF and COX-2 reduction in SCT^−/−^ mice. (**A**) Low and high magnification of cross-sectional and longitudinal images showing the wall morphology changes as small airway remodeling in SCT^−/−^ mice. (**B**) Immunohistochemical staining of VEGF showing protein level reduction at the corresponding time points. (**C**) VEGF was reduced in the lung tissue and BAL fluid in SCT^−/−^ mice (n = 6/group). (**D**) Immunohistochemical staining of COX-2 indicating lower protein content in SCT^−/−^ mice. COX-2 is known to be directly related to VEGF level and inhibits apoptosis (n = 6/group)^27^.

**Figure 7:**
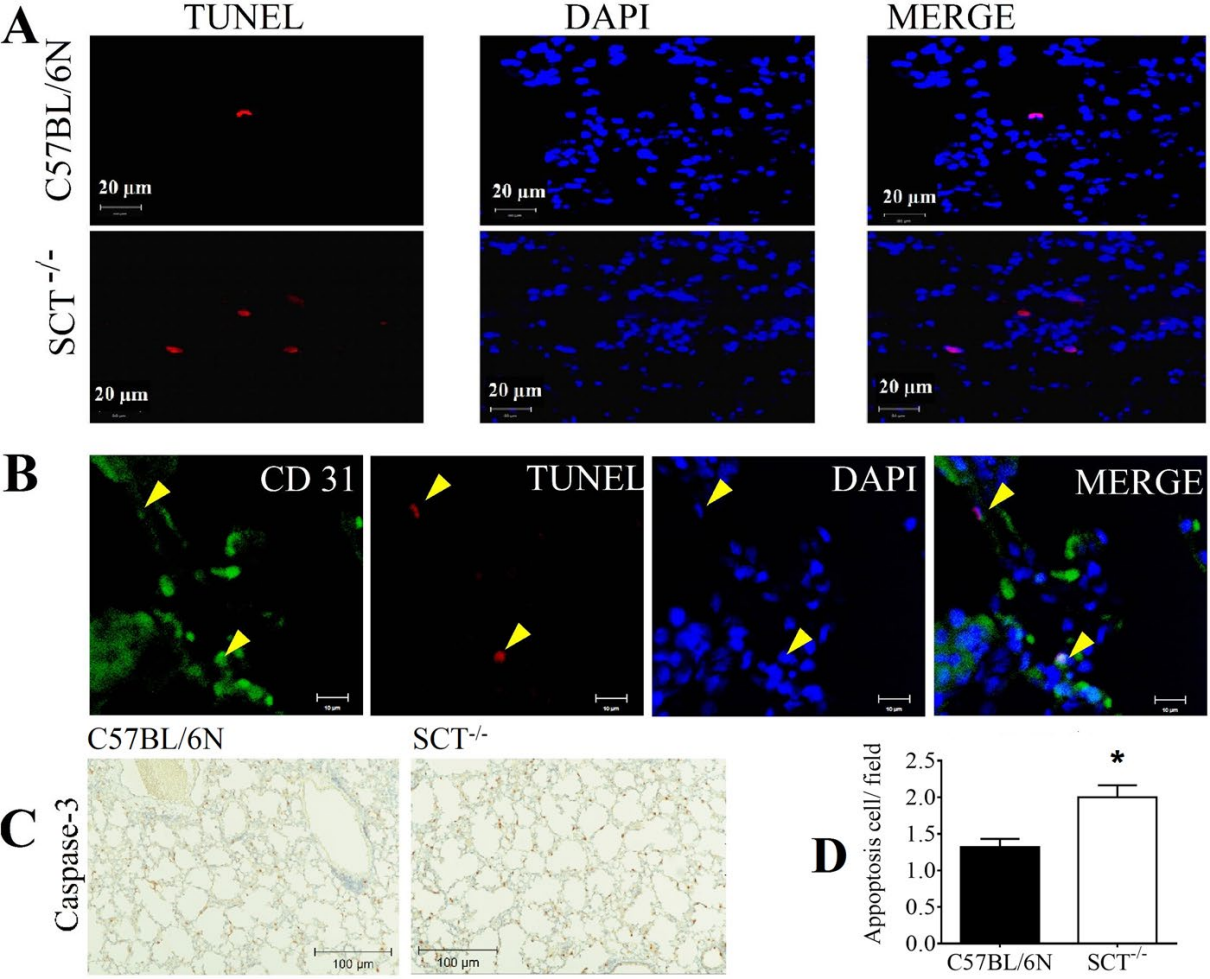
Increased apoptosis in the lungs of SCT^−/−^ mice. (**A**) TUNEL staining of the lungs shows increased apoptosis in SCT^−/−^ mice compared with C57BL/6N mice (**B**) TUNEL/CD31 double staining in the lung sections of SCT^−/−^ mice showing an apoptotic endothelial cell [yellow arrow heads] (n = 6). (**C**) Caspase-3, an indicator of apoptosis, staining from control and SCT^−/−^ mice lungs showing higher caspase-3 positive cells in SCT^−/−^ lungs (n = 5/group)^28^. (**D**) Increased apoptotic cell count in SCT^−/−^ mice (from 6 different sections from each mouse, n = 6/group) *p < 0.05.

Apart from arterial wall remodelling, small airway remodelling was also found in SCT^−/−^ mice. From the H&E staining, although the columnar cells were still present in SCT^−/−^ mice, the morphology was significantly changed. Thinner bronchiolar epithelium with the loss or deformation of the shape of native club cells was observed in SCT^−/−^ mice compared to control mice (Fig. 6A). Next, we analysed the VEGF expression level in these lung sections because VEGF itself is primarily localized in bronchiolar and alveolar epithelial cells and is responsible for angiogenesis^26^. Immunostaining revealed a reduction in VEGF expression in the lung tissue and bronchoalveolar lavage (BAL) of SCT^−/−^ mice at 6, 9 and 12 months of age (Fig. 6B). Meanwhile, immunostaining of COX-2, a protein that is known to be directly related to VEGF level and inhibits apoptosis^27^, was performed and also found a much lower COX-2 expression in SCT^−/−^ mice compared to that in control mice (Fig. 6D). Since reduced COX-2 expression in the lungs of SCT^−/−^ mice may indicate an increase in apoptosis, TUNEL staining and double staining of TUNEL/CD31 were carried out. Both stainings showed increased apoptosis and apoptotic ECs in the lungs of SCT^−/−^ mice (Fig. 7A,B,D). A consistent result was also given according to the immunostaining of caspase-3, an indicator of apoptosis^28^, in which the staining revealed higher caspase-3-positive cells in SCT^−/−^ lungs compared to control mouse lungs (Fig. 7C). Taken together, the changes in lung morphology and increased apoptosis in the lungs of SCT^−/−^ mice suggested that SCT^−/−^ mice exhibit small airway remodelling.

### Molecular changes in lung tissue hormones and protein in SCT^−/−^ mice

Apart from the observed deteriorated lung tissues in SCT^−/−^ mice, related lung tissue hormones and proteins were also analysed to study the underlying SCT mechanism. Significantly elevated transcript levels of tumour necrosis factor alpha (TNF-α), ET-1 and endothelin-A receptor (ETAR) (3.55-fold, 3.21-fold, and 2.3-fold *vs*. control). Meanwhile, VEGF, prostaglandin-endoperoxide synthase-2 (PTGS-2), vasoactive intestinal polypeptide type-1 receptor transcript level (VPAC1R) and transforming growth factor-beta (TGF-β) were downregulated in the lung tissues of SCT^−/−^ mice (Fig. 4D).

### Long-term SCT treatment can prevent heart and lung pathologies

**Figure 8:**
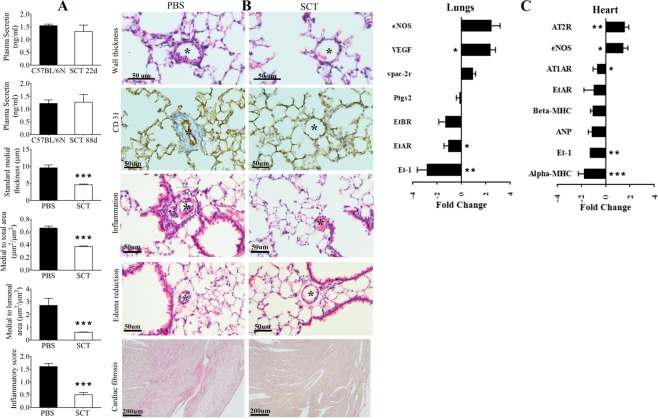
SCT treatment reduces the pathologies in the lungs and heart along with the gene expression changes. * indicates lumen of an artery. (**A**) Plasma SCT levels at day-22 and day-88 in SCT treated SCT^−/−^ mice are comparable with the SCT levels of same age C57BL/6N mice. The reduction of arterial wall thickness was observed in the SCT treatment group (n = 6/group; ***p < 0.001). The reduction of the inflammatory score was observed in the lungs of SCT-treated SCT^−/−^ mice. (**B**) The images show the reduction of arterial wall thickness, endothelial cell proliferation, perivascular inflammation and adventitial expansion and cardiac fibrosis in SCT-treated SCT^−/−^ mice. (**C**) Gene expression changes in the lungs and hearts of SCT-treated SCT^−/−^ mice compared with the PBS-treated SCT^−/−^ mice.

Improper management of SHT and PAH will result in fatal consequences, including kidney failure, congestive heart failure and stroke^3^. Given that SCT has an important cardiovascular and pulmonary effect, long-term SCT treatment was carried out to test its possibility of treating hypertension. In this article, the effect of long-term SCT treatment was tested in 3-month-old SCT^−/−^ mice using a mini-osmotic pump, and successful infusion was confirmed by showing no significant difference in SCT levels in all SCT-infused SCT^−/−^ groups compared to similar age control mice on day 22 and day 88 of treatment (Fig. 8A). Blood pressure measurements for SCT supplemented animals were not carried out and this constitutes a key limitation in the current study. After long-term SCT treatment in SCT^−/−^ mice, the medial width (standard medial thickness), medial area to total area ratio, medial area to luminal area ratio and inflammatory score in the lungs were significantly reduced in the SCT-replacement group (Fig. 8A). As shown in the histomorphology analysis of the heart and lungs, arterial wall thickening, endothelial cell proliferation, perivascular inflammation and adventitial expansion, and cardiac fibrosis were all reduced in the SCT-treated group (Fig. 8B). In the lungs of SCT-treated SCT^−/−^ mice, ET-1 and ETAR transcript levels were significantly downregulated (0.37 ± 0.08-fold and 0.68 ± 0.07-fold *vs*. control), while VEGF transcript levels were upregulated (2.27 ± 0.35-fold). As for the heart of SCT-treated SCT^−/−^ mice, ET-1 and alpha-MHC transcript levels were downregulated (0.64 ± 0.03-fold and 0.54 ± 0.08-fold *vs*. control), while the eNOS transcript level was upregulated (1.65 ± 0.23-fold *vs*. control) (Fig. 8C). These results collectively suggest that long-term SCT treatment can improve heart and lung conditions due to hypertension, correction of related gene expression changes.

## Discussion

In this report, it is revealed that SCT^−/−^ mice exhibit both systemic and pulmonary hypertension with damage in the heart and lungs, in addition to previously reported cardiovascular and pulmonary effects*. The systemic hypertension in SCT^−/−^ mice could be due to dysregulation of the RAAS with lowered VEGF and NO levels and higher aldosterone levels because of SCT deficiency. SCT could upregulate VEGF production in epithelial cells of the bile ducts^17^ and exert coronary vasodilation via augmented endothelial release of NO^14,15,29^. Our results showed a significant reduction in plasma VEGF and NO levels in SCT^−/−^ mice. VEGF can stimulate blood vessel formation and trigger NO production to regulate vasodilation^30^. NO has acute vasodilation properties^31^ and NO reduction can cause systemic hypertension^32,33^. As a result, the significant decrease in VEGF and NO in SCT^−/−^ mice could avoid vasodilation and result in continuous vasoconstriction, which then increased SBP, DBP and MAP and led to LV and RV pathologies. The damage to cardiac tissue can be due to the pressure overload in the pulmonary and systemic circulations or from the heart itself. As a result, cardiac fibrosis was due to the continual increase in blood pressure and heavy workload in cardiac muscles. Moreover, these pathologies are mainly localized in the RV and IVS of the heart of SCT^−/−^ mice, likely due to high pulmonary arterial blood pressure.

Another contributing factor to the systemic hypertension in SCT^−/−^ mice is the higher aldosterone level because excess aldosterone can cause systemic hypertension in humans^34,35^ and animals^36^ as well as favour cardiac apoptosis and fibrosis^37,38^. However, the higher aldosterone level in SCT^−/−^ mice may be due to the reduction of NO because it can inhibit aldosterone production^39^. Meanwhile, the genes related to myocardial fibrosis, namely, ET-1, ET-1, AT1AR and AT1BR expression^40^, were significantly increased with the augmented cardiac aldosterone. In contrast, eNOS expression was significantly decreased in the hearts of SCT^−/−^ mice, which may induce and enhance myocardial fibrosis^41^. The increased cardiac VEGF level could be the result of ET-1 overexpression^42^ and increased cardiac aldosterone^43^. These may be the combined cause of cardiac fibrosis and apoptosis. Furthermore, expression of cardiac hypertrophic marker genes including beta-MHC, alpha-MHC, and alpha-SKA are all found to be high in the heart of SCT^−/−^ mice^44^.

We observed robust arterial remodelling, perivascular inflammation, and adventitial expansion in the lungs of SCT^−/−^ mice, which are associated with pulmonary hypertension. Numerous human^45^ and animal studies^46^ had reported that altered immunity and inflammation are the causes of PAH^47^, and reversion of arterial remodelling can reduce pulmonary arterial pressure^48^. In this study, we found that the expression of the pro-inflammatory cytokine TNF-alpha, an inflammation marker^49^, was significantly increased in the lungs of SCT^−/−^ mice, which would be a contributing factor for PAH in SCT^−/−^ mice with reduced VEGF^50^ and increased ET-1 expression^51^. Furthermore, the small airway remodelling found in SCT^−/−^ mice can also be the reason for the inflammation reaction in the lungs^52,53^. The disruption of the pulmonary bronchiolar epithelium with the loss or deformation of the shape of native club cells observed in SCT^−/−^ mice may be due to the consequences of SCT deficiency on Cl^−^ and HCO_3_^−^ secretion in the lungs, as this is necessary for airway surface liquid (ASL) to maintain a healthy epithelium in the lungs^12^. This epithelium defect also contributes to the reduction of TGF-beta, iNOS, and VEGF expression in bronchiolar epithelium^54–56^ found in SCT^−/−^ mice. It was reported that the VEGF ameliorates PAH^57^ and that VEGFR inhibition can cause PAH^58,59^; as such, the bronchiolar epithelium depletion may cause VEGF and VEGFR reduction and then favour the occurrence of PAH in SCT^−/−^ mice^54^. Apart from VEGF/R deficiency, excess aldosterone and NO deficiency could also be contributing factors for PAH in SCT^−/−^ mice because aldosterone can decrease NO levels in the lungs and promote PAH^60^.

The EC cell apoptosis and hyperproliferation in SCT^−/−^ lungs are important in the pathogenesis of PAH^61^. Caspase-3, which is activated in apoptotic cells^28^, was increased in the lungs of SCT^−/−^ mice, while the lower VEGF level may decrease PTGS-2 (COX-2) release and result in defects in prostaglandin synthesis^62^, and further lead to apoptosis and neutrophil migration in the lungs^63^. In addition, increased ET-1 and aldosterone levels in the lungs of SCT^−/−^ mice can promote pulmonary artery proliferation^64^ and perivascular fibrosis^65^, where these two consequences can cause sustained pulmonary hypertension in SCT^−/−^ mice. However, currently, no *in vivo* or *in vitro* experiments directly support this theory, and further investigation should be carried out to confirm this hypothesis.

Blood pressure (BP) reduction and organ damage prevention are the major goals for all hypertensive drugs. In our study, short- and long-term SCT treatments seemed to be beneficial for the SCT^−/−^ mice. The one-week-long SCT treatment can reduce plasma aldosterone, renin and the aldosterone to renin ratio, while the 3-month-long SCT treatment can prevent the development of heart and lung pathologies in SCT^−/−^ mice. In the long-term SCT-treated SCT^−/−^ mice, arterial wall thickening, perivascular adventitial expansion and inflammatory cell proliferation were all reduced. The reduction of perivascular adventitial expansion and inflammation could be due to reduced plasma aldosterone, as aldosterone can promote vascular inflammation, and inhibition of aldosterone would be beneficial^66^. However, here, we can only provide the information that excess aldosterone is involved in the pathophysiologic mechanisms of SCT deficiency, but we cannot conclude yet whether this is primarily due to SCT deficiency or is a secondary contribution resulting from the cardiac pathologies. Aside from the histopathological changes, long-term SCT treatment on SCT^−/−^ mice can also reduce ET-1 and ETAR expression and increase VEGF and eNOS expression in the lungs. Similarly, ET-1 and AT1AR expression were decreased, while eNOS and AT2R expression were significantly increased in the heart.

This study suggested that SCT can be an essential hormone for the cardiovascular and pulmonary systems in humans since SCT deficiency can result in pulmonary and systemic hypertension in mice with fibrosis in the heart and lungs. Our findings can also explain the reduced SCT and VEGF and increased aldosterone levels in chronic heart failure patients to a certain extent^21,22^. Investigating the SCT level in patients with pulmonary and systemic hypertension can provide interesting information, such as whether SCT deficiency has a role in pulmonary and systemic hypertension in humans.

## Methods

### Experimental animals

SCT^−/−^ mice were generated as per previously described methods^18^. The studies used 3, 6, 9 and 12-month-old male SCT^−/−^ mice with C57BL/6N mice as controls. SCT^+/−^ mice were backcrossed with female C57BL/6N mice, and all experiments were carried out using at least N10 generation mice.

### Experimental design and studies

Procedures and animal handling were in accordance with the protocols approved by the Committee on the Use of Live Animals in Teaching and Research of the University of Hong Kong, Animal Subjects Ethics Sub Committee of the Hong Kong Polytechnic University and Declaration of Helsinki. Anatomical and histopathological studies, echocardiography and hemodynamic measurements, real-time PCR, vascular endothelial growth factor (VEGF) measurements, *in situ* cell death detection, plasma hormone analysis and SCT replacement therapy were performed. The guidelines for good laboratory animal practice were applied whenever the mice were sacrificed for experiments.

### Anatomical study

Right ventricle to left ventricle plus septum ratio [RV/(LV + S)] was used to compare the RV hypertrophy between SCT^−/−^ and C57BL/6N^67^. After euthanasia with 5% isoflurane in 2 L/min oxygen flow in the induction chamber, the chest wall was opened, and heart and lungs of pre-heparinized mice were inspected for visible abnormalities. The hearts were taken out, thoroughly cleaned in normal saline, connecting vessels and atria were removed, and the heart was blotted dry on lint-free paper towels^68^. The RV was thoroughly excised from LV and IVS and weighed with a precision scale.

### Histopathological study

Hearts, of heparinized and anesthetized mice, were stopped at diastole with slow infusion of ice-cold 30 mM KCl solution into the posterior basal aspect of ventricles^69^. The hearts and lungs were taken out and washed in PBS. Neutral Buffered Formalin (NBF) was slowly injected from the trachea into lungs and, from its apex, into the heart. Both tissues were fixed in the 20 times weight to volume NBF overnight and embedded in paraffin. Sections (5 μm) were stained with H&E by an ST5020 multi-stainer (Leica Biosystem) and picrosirius red collagen staining (Poly Sciences Int.) for histopathology and fibrosis analysis respectively. Arteries in size range 50–100 μm were used for comparing changes in wall thickness, perivascular fibrosis, adventitial expansion formation and perivascular inflammatory scoring. Arteries close to bronchi or terminal bronchioles (∼50 μm diameter) were selected for measurement of the total area (µm^2^), luminal area (µm^2^) and the inner circumference of arteries (µm) (Supplementary Fig. S2). The difference between total and luminal area was calculated as medial area (µm^2^). For fibrosis area analysis, Image J software was used and performed as previously described (4 separate vessels/mouse, 6 mice/group)^70^. Standard medial thickness was calculated by the ratio of medial area to inner circumference while average vessel and the diameter was obtained from total area measurements by the SPOT ADVANCED software (Diagnostic Instruments)^71^. For inflammation, score “0” was given for no inflammation, score “1” was given for the occasional cuffing with inflammatory cells, score “2” was given if most bronchi or vessels were surrounded by a 1–5 cell thick layer of inflammatory cells, and score “3” was given if most bronchi or vessels were surrounded by a more than 5 cells thick layer of inflammatory cells as previously described^72^. Immunohistochemical (IHC) staining was performed using paraffin-embedded left lung section and antibodies for CD31 (1:50 dilution), VEGF (1:50 dilution), COX-2 (1:300 dilution) (Abcam) and Caspase-3 (1:800 dilution, Cell Signaling Technologies)^73^.

### Echocardiography and hemodynamic measurements

Mice were anesthetized with 3% isoflurane (induction) followed by 1–1.5% isoflurane (maintenance) with 2 L/min oxygen flow, and high frequency echocardiography was performed by Vevo 2100 System with MS550D 22–55 MHz transducer (FUJIFILM VisualSonics Inc.). Blood flow measurements were performed when the heart rate was between ∼400 to 500 bpm to mimic near physiological conditions and consistency. Ventricle and septum morphologies, pulmonary arterial diameters and blood flow were compared. Two dimensional (B mode), measurement (M mode), pulsed wave Doppler and color Doppler methods were used for data acquisition, and results were analyzed with accompanied Vevo 2100 software and DICOM 3.0 software^74^. Pressure measurements were performed using HD-X11 telemetry transmitters (Data Sciences International) according to manufacturer’s protocol. For right ventricular systolic pressure (RVSP) measurement, the pressure probe was inserted through the right jugular vein and advanced to the RV for 20–30 minutes recording^75^. For arterial blood pressure (BP) measurement, the probe was inserted into the left common carotid artery and advanced into the aortic arch for 48 h continuous recording 8 days after post operation^76^.

### Real-time PCR and VEGF measurements

The right lung was homogenized in TRIzol (Invitrogen) for RNA extraction^77^ and quantitative real-time PCR (ABI Prism 7500, Applied Biosystems) with the SYBR PCR Master Mix kit (Applied Biosystems) and gene specific primers (Supplementary Table S1). Results were normalized to GAPDH, and relative gene expression was calculated using the delta-delta CT method^78^. Bronchoalveolar lavage fluid (BALF) was obtained as described^79^ and VEGF levels were measured using an ELISA kit (R&D Systems).

### *In situ* cell death detection

TMR red (Roche Applied Science) was used for *in situ* TUNEL staining and TUNEL/CD31 double staining as described^57^. Briefly, heat-induced retrieval (citrate buffer) was performed prior to TUNEL, followed by labeling with anti-CD31 and anti-rabbit FITC antibody. Positive (DNase-treated section) and negative controls (terminal transferase omitted) were run in parallel. Apoptotic cells in 5 fields per slide, 6 slides for mouse, and 6 mice per genotype were considered for calculations.

### Plasma, serum and tissue homogenate analysis

Blood collection, serum and plasma extraction, and tissue homogenate preparations were carried out as per the manufacturers’ protocol. Plasma hormone levels were measured using ELISA kits [Secretin, Angiotensin II and Vasopressin (Phoenix Pharmaceuticals Inc.), Renin LS F508 kit (Life Span BioSciences, Inc.), Aldosterone ELISA Kit (Enzo Life Science)], VEGF Quantikine ELISA Kit (R&D Systems), NO Assay Kit (Abcam) and plate reader at the respective wavelengths. Results were analyzed and compared between groups.

### SCT treatment

SCT^−/−^ mice were treated with PBS or SCT (2.5 nmol/kg/day) by intraperitoneal implantation of 2004 model mini-pumps (Alzet) as described^73^. The mice were treated with SCT or PBS for a week, a month and three months depending on the experimental needs. For the 3-month-long treatment, the pumps were replaced every 22^nd^ day for a total of 88 days followed by euthanasia and pathophysiological study in lungs and hearts. Plasma levels of SCT were measured, using ELISA kit (Phoenix Pharmaceuticals Inc.), at 22^nd^ day and 88^th^ day of implantation to confirm successful infusion.

### Statistical analysis

All data are shown as means ± SEM. The deviations between groups were analyzed using Prism 6.0 software (GraphPad Software Inc.). Data were analyzed either using Student’s *t*-test or 1-way ANOVA, followed by Dunnett’s test. All data analysis was conducted under blinded conditions. Differences were considered significant if *p* < 0.05.

## Supplementary information

Supplementary video S1

Supplementary video S2

Supplementary information

## Data Availability

The data generated and/or analyzed in the current study are included in this article (and its Supplementary Information Files) or available from the corresponding author on reasonable request.
