# Supplementary material for: Loss of secretin results in systemic and pulmonary hypertension with cardiopulmonary pathologies in mice
Source: Sci Rep. 2019 Oct 2;9:14211. doi: 10.1038/s41598-019-50634-x (PMC6775067; doi:10.1038/s41598-019-50634-x)
Supplement: Supplementary file 3 — Supplementary information [file 41598_2019_50634_MOESM3_ESM.pdf]

(Supplementary Material Online)

Loss of Secretin results in systemic and pulmonary hypertension with cardiopulmonary pathologies in mice

Aung Moe Zaw[1]†, Revathi Sekar[1]†, Sarah Mak Oi Kwan[1], Helen K.W. Law2*, Billy Kwok Chong Chow1*.

1 School of Biological Sciences, The University of Hong Kong, Hong Kong, China.

2 Department of Health Technology and Informatics, Faculty of Health and Social Sciences, The Hong Kong Polytechnic University, Hong Kong, China.

† These authors contributed equally to this work.

*Corresponding Authors

Correspondence and requests for materials should be addressed to

Helen K. W. Law: helen.law@polyu.edu.hk and Billy Kwok Chong Chow: bkcc@hku.hk.

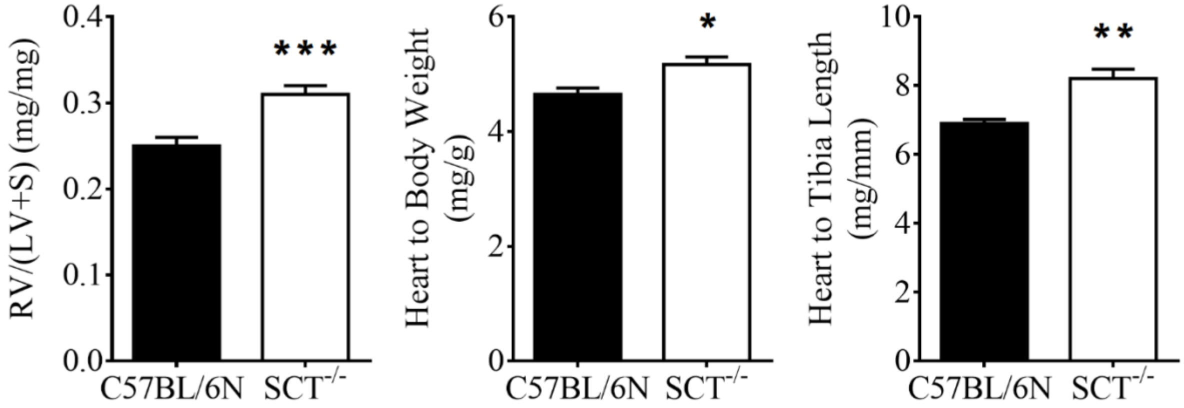

Supplementary Figure S1. Heart weight ratios were increased in 3-mold-old SCT$^{-/-}$ mice. The right ventricle to left ventricle plus septum ratio, heart to body weight ratio and heart to tibia length ratios were significantly increased in 3-month-old SCT$^{-/-}$ mice compared with control mice. (n = 7-8/group, * = $p < 0.05$, ** = $p < 0.01$, *** = $p<0.001$).

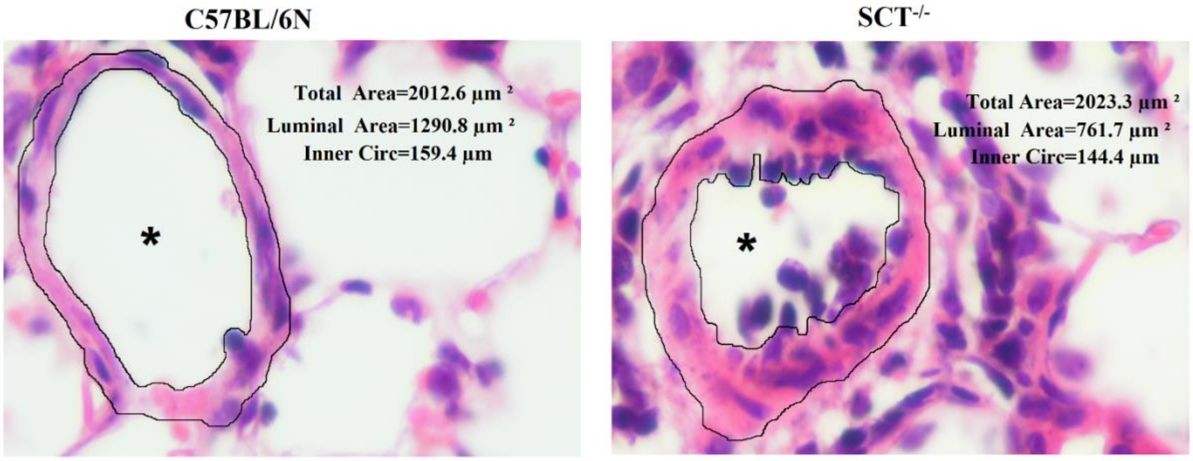

Supplementary Figure S2. Representative images of the arterial thickness measurement in the lungs of 6-month-old C57BL/6N and SCT$^{-/-}$ mice. * represents the lumen of the artery. Although the total areas are similar in both mice, the luminal area and inner circumference are smaller in the SCT$^{-/-}$ mouse compared with the C57BL/6N mouse.

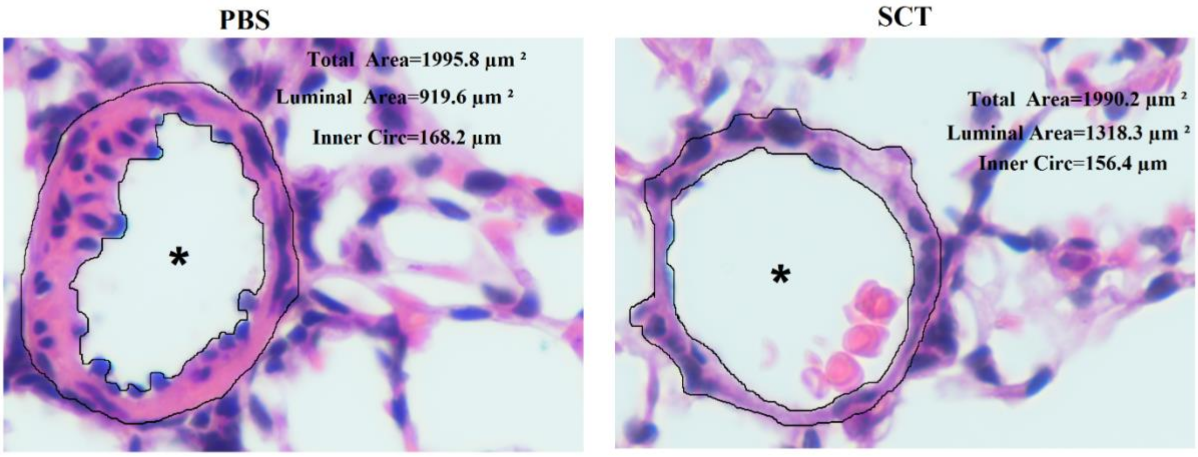

Supplementary Figure S3. Representative images of the arterial thickness measurement in 3-month-long PBS and SCT treated SCT$^{-/-}$ mice. * represents the lumen of the artery. Although the total areas are similar in both mice, the wall thickness is reduced in SCT treated SCT$^{-/-}$ mouse compared with PBS treated SCT$^{-/-}$ mouse.

Supplementary Table S1. Primer sequence

Primer	Sequence
glyceraldehyde 3-phosphate dehydrogenase (GAPDH)	TGTGTCCGTCGTGGATCTGA
	TTGCTGTTGAAGTCGCAGGAG
vascular endothelial growth factor (VEGF)	CAGTTCGAAGGAAAGGGAAAGG
	CACGTCTGCGGATCTTGGAC
Endothelin-1 (ET-1)	GCCACAGACCAGGCAGTTAGA
	CACCAGCTGCTGATAGATACACTTC
Endothelial nitric oxide synthase (eNOS)	CCAGTGCCCTGCTTCATC
	GCAGGGCAAGTTAGGATCAG

Endothelin type-A receptor (ETAR)	GCTCGTTCCCTCTTCACTTAAGC
	TCATGGTTGCCAGGTTAATGC
Endothelin type-B receptor (ETBR)	CATGCGCAATGGTCCCAATA
	GCTCCAAATGGCCAGTCCTC
Tumor necrosis factor- alpha (TNF-α)	AGTTCCCAAATGGCCTCCC
	GCTACGACGTGGGCTACAGG
Prostaglandin-endoperoxide synthase-2 (PTGS-2)	TGGGCCATGGAGTGGACTT
	CCACCAATGACCTGATATTTCAAT
Interleukin-6 (IL-6)	TCTATACCACTTCACAAGTCGGA
	GAATTGCCATTGCACAACTCTTT
Interleukin-6 receptor (IL-6R)	ATCCTCTGGAACCCCACAC
	GAACTTTCGTACTGATCCTCGTG
Transforming growth factor-beta (TGF-β)	TTCCGCTGCTACTGCAAGTCA
	GGGTAGCGATCGAGTGTCCA
B-cell lymphoma-2 (BCL-2)	GTGTTCCATGCACCAAGTCCA
	AGGTACAGGCATTGCCGCATA
Bone morphogenetic protein receptor-2 (BMPR-2)	GAGCCCTCCCTTGACCTG
	GTATCGACCCCGTCCAATC
Vascular endothelial growth factor-receptor	CCTACCTCACCTGTTTCCTGTATG

type2 (VEGF-R2)	ACCATCCCACTGTCTGTCTGG
Vasoactive intestinal type-1 receptor (VPAC-1R)	AACTTTAAGGCCCAGGTGAAAT
	CCTGCACCTCGCCATTG
Vasoactive intestinal type-2 receptor (VPAC-2R)	
	TGAGCCCAAGATGAGGGC
	GTTCACTCACCCGCACCAG
Pituitary adenylate cyclase activating polypeptide -1r (PAC-1R)	CAAGAAGGAGCAAGCCATGTGC
	CATCGAAGTAATGGGGGAAGG
Atrial natriuretic peptide (ANP)	ATTGACAGGATTGGAGCCCAGAGT
	TGACACACCACAAGGGCTTAGGAT
Brain natriuretic peptide (BNP)	GTTTGGGCTGTAACGCACTGA
	GAAAGAGACCCAGGCAGAGTCA
Insulin like growth factor-1 (IGF-1)	TGCTCTTCAGTTCGTGTG
	ACATCTCCAGTCTCCTCAG
Alpha-myosin heavy chain (α-MHC)	ATCATTCCCAACGAGCGAAAG
	AAGTCCCCATAGAGAATGCGG
Beta-myosin heavy chain (β-MHC)	ATGTGCCGGACCTTGGAAG
	CCTCGGGTTAGCTGAGAGATCA
Alpha-skeletal actin (α-SKA)	TGAGACCACCTACAACAGCA

	CCAGAGCTGTGATCTCCTTC
Angiotensin-2 receptor (AT-2R)	TTATTACCTGCATGAGTGTCGATAGG
	AGATGCTTGCCAGGGATTCC
Angiotensin-1a receptor (AT-1AR)	GCGGTCTCCTTTTGATTTCC
	CAAAGGGCTCCTGAAACTTG
Angiotensin-1b receptor (AT-1BR)	TGTTGCTTCCTTGTCCCTTG
	TATTTTCCCCAGAGCAAAGC

Supplementary Table S2. Expression of gene transcript levels in heart of $SCT^{-/-}$ mice after 3-6 month SCT infusion compared with PBS infused mice

**Gene expression in heart after SCT infusion at 3-6 months**	**Expression fold change over PBS treated $SCT^{-/-}$ mice**
Atrial natriuretic peptide (ANP)	0.67±0.06 (p=0.0816)
Brain natriuretic peptide (BNP)	1.34±0.54
Insulin like growth factor-1 (IGF-1)	1.05±0.23
Alpha-myosin heavy chain (α-MHC)	0.54±0.08***
Beta-myosin heavy chain (β-MHC)	0.69±0.04 (p=0.0754)
Alpha-skeletal actin (α-SKA)	1.00±0.37
Endothelial nitric oxide synthase (eNOS)	1.65±0.23*
Endothelin-1 (ET-1)	0.64±0.03**
Endothelin type-A receptor (ETAR)	0.70±0.17 (p=0.0820)
Endothelin type-B receptor (ETBR)	1.38±0.30

Angiotensin-1a receptor (AT-1AR)	0.78±0.08*
Angiotensin-1b receptor (AT-1BR)	0.88±0.09
Angiotensin-2 receptor (AT-2R)	1.74±0.20**

Internal control: GAPDH expression. 6 months old mice; n=6; *, p<0.05; **, p<0.01; ***, p<0.001.

Supplementary Table S3. Left ventricular echocardiographic parameters.

**3MO (n=7/group)**	**C57BL/6N**	**SCT$^{-/-}$**
**EF**	59.12±1.22	61.32±4.29
**FS**	30.88±0.83	32.76±3.04
**LV Mass AW**	98.75±2.16	84.14±4.42*
**LV Mass AW (Corrected)**	79.00±1.73	67.31±3.54*
**LV Vol;d**	67.14±5.51	53.43±5.52
**LV Vol;s**	27.55±2.61	21.59±3.97
**LVAW;d**	0.79±0.06	0.78±0.04
**LVAW;s**	1.23±0.06	1.24±0.08
**LVID;d**	3.91±0.14	3.54±0.17
**LVID;s**	2.70±0.11	2.40±0.19
**LVPW;d**	0.67±0.02	0.67±0.02
**LVPW;s**	1.05±0.02	1.03±0.05

(*, p<0.05)

**6MO (n=7-9/group)**	**C57BL/6N**	**SCT$^{-/-}$**

**EF**	57.75±3.52	62.03±4.87
**FS**	30.43±2.31	34.46±4.20
**LV Mass AW**	127.12±12.94	98.24±4.23*
**LV Mass AW (Corrected)**	101.70±10.35	78.59±3.38
**LV Vol;d**	80.53±9.00	65.99±6.22*
**LV Vol;s**	35.89±6.96	27.00±5.04
**LVAW;d**	0.90±0.03	0.81±0.04
**LVAW;s**	1.23±0.07	1.27±0.06
**LVID;d**	4.21±0.19	3.87±0.16
**LVID;s**	2.95±0.23	2.57±0.24
**LVPW;d**	0.67±0.03	0.65±0.03
**LVPW;s**	1.10±0.05	1.07±0.09

(*, $p<0.05$)

**9MO (n=5/ group)**	**C57BL/6N**	**$SCT^{-/-}$**
**EF**	62.30±3.00	61.06±3.10
**FS**	33.27±2.09	32.35±2.22
**LV Mass AW**	124.97±5.65	109.18±5.51
**LV Mass AW (Corrected)**	99.97±4.52	87.35±4.41
**LV Vol;d**	66.61±6.71	61.26±4.61
**LV Vol;s**	26.08±4.24	24.38±3.06
**LVAW;d**	1.02±0.09	0.89±0.07

**LVAW;s**	1.50±0.11	1.31±0.09
**LVID;d**	3.89±0.17	3.77±0.12
**LVID;s**	2.61±0.19	2.56±0.15
**LVPW;d**	0.72±0.04	0.74±0.03
**LVPW;s**	1.17±0.09	1.13±0.06

(*, p<0.05)

**12MO (n=5-6/group)**	**C57BL/6N**	**SCT$^{-/-}$**
**EF**	61.88±4.13	57.85±2.94
**FS**	33.46±3.10	30.28±1.97
**LV Mass AW**	152.85±8.50	128.43±9.83
**LV Mass AW (Corrected)**	122.28±6.80	102.75±7.86
**LV Vol;d**	75.60±9.41	74.22±5.32
**LV Vol;s**	29.93±5.37	31.70±4.01
**LVAW;d**	1.01±0.05	0.88±0.02
**LVAW;s**	1.52±0.08	1.31±0.04
**LVID;d**	4.09±0.21	4.09±0.12
**LVID;s**	2.74±0.23	2.86±0.14
**LVPW;d**	0.89±0.06	0.77±0.04
**LVPW;s**	1.37±0.11	1.15±0.04

(*, p<0.05)

EF	Ejection Fraction
FS	Fractional Shortening
LV Mass AW	Left Ventricle Mass Anterior Wall
LV Mass AW (Corrected)	Left Ventricle Mass Anterior Wall (Corrected)

LV Vol;d	Left Ventricle Volume; diastole
LV Vol;s	Left Ventricle Volume; systole
LVAW;d	Left Ventricle Anterior Wall; diastole
LVAW;s	Left Ventricle Anterior Wall; systole
LVID;d	Left Ventricle Internal Dimension; diastole
LVID;s	Left Ventricle Internal Dimension; systole
LVPW;d	Left Ventricle Posterior Wall; diastole
LVPW;s	Left Ventricle Posterior Wall; systole

Supplementary video S1. Representative short axis biventricular views of 3-month-old and 6-month-old C57BL/6N (C57 in the video) mouse and SCT$^{-/-}$ mouse. 3-month-old SCT$^{-/-}$ mouse had a thicker right ventricular wall and 6-month-old SCT$^{-/-}$ mouse had thinner RV wall, likely due to fibrosis with myocardium loss and dilation. However, both SCT$^{-/-}$ mice tracing showed thicker interventricular septum than control C57BL/6N mice.

Supplementary video S2. Representative short axis biventricular views of 9-month-old and 12-month-old C57BL/6N (C57 in the video) mouse and SCT$^{-/-}$ mouse. 9-month-old SCT$^{-/-}$ mouse had thinner RV wall and thicker white with disrupted IVS. 12-month-old SCT$^{-/-}$ mouse had irregular RV wall movement with deformed RV, thin IVS at the right side of the wave direction line with thin and bulging of one side of the RV wall (arrow).
